# Development of CT-Based Imaging Signature for Preoperative Prediction of Invasive Behavior in Pancreatic Solid Pseudopapillary Neoplasm

**DOI:** 10.3389/fonc.2021.677814

**Published:** 2021-05-17

**Authors:** Wen-peng Huang, Si-yun Liu, Yi-jing Han, Li-ming Li, Pan Liang, Jian-bo Gao

**Affiliations:** ^1^ Department of Radiology, The First Affiliated Hospital of Zhengzhou University, Zhengzhou, China; ^2^ Pharmaceutical Diagnostics, General Electric Company (GE) Healthcare, Beijing, China

**Keywords:** pancreatic solid pseudopapillary neoplasm, computed tomography, invasiveness, radiomics, diagnosis

## Abstract

**Purpose:**

It is challenging for traditional CT signs to predict invasiveness of pancreatic solid pseudopapillary neoplasm (pSPN). We aim to develop and evaluate CT-based radiomics signature to preoperatively predict invasive behavior in pSPN.

**Methods:**

Eighty-five patients who had pathologically confirmed pSPN and preoperative contrasted-enhanced CT imaging in our hospital were retrospectively analyzed (invasive: 24; non-invasive: 61). 1316 radiomics features were separately extracted from delineated 2D or 3D ROIs in arterial and venous phases. 200% (SMOTE) was used to generate balanced dataset (invasive: 72, non-invasive: 96) for each phase, which was for feature selection and modeling. The model was internally validated in the original dataset. Inter-observer consistency analysis, spearman correlation, univariate analysis, LASSO regression and backward stepwise logical regression were mainly applied to screen the features, and 6 logistic regression models were established based on multi-phase features from 2D or 3D segmentations. The ROC analysis and Delong’s test were mainly used for model assessment and AUC comparison.

**Results:**

It retained 11, 8, 7 and 7 features to construct 3D-arterial, 3D-venous, 2D-arterial and 2D-venous model. Based on 3D ROIs, the arterial model (AUC: 0.914) performed better than venous (AUC: 0.815) and the arterial-venous combined model was slightly improved (AUC: 0.918). Based on 2D ROIs, the arterial model (AUC: 0.814) performed better than venous (AUC:0.768), while the arterial-venous combined model (AUC:0.893) performed better than any single-phase model. In addition, the 3D arterial model performed better than the best combined 2D model. The Delong’s test showed that the significant difference of model AUC existed in arterial models in original dataset (p = 0.019) while not in arterial-venous combined model (p=0.49) as comparing 2D and 3D ROIs.

**Conclusion:**

The arterial radiomics model constructed by 3D-ROI feature is potential to predict the invasiveness of pSPN preoperatively.

## Introduction

Pancreatic solid pseudopapillary neoplasm (pSPN) is a rare low-grade malignant tumor, accounting for 1%–2% of pancreatic exocrine tumors. It usually occurs in women under the age of 40. Its clinical manifestations are not typical. Most of them come to the hospital with asymptomatic physical examination, abdominal pain or touching abdominal mass as the main complaint. Laboratory examination is of little help in its diagnosis, and the final diagnosis depends on the immunohistochemical results of postoperative pathology (1–3). A 2018 Chinese multicenter retrospective study showed that pSPN accounted for 31.7% of all resected pancreatic cystic tumors (4). According to the classification criteria of digestive system tumors of the World Health Organization, SPN is defined as invasive when the tumor obviously breaks through the capsule or invades the peripancreatic tissue, surrounding organs and blood vessels, vascular invasion, peripheral nerve invasion, lymph node metastasis and distant metastasis (5, 6). Surgery is the only treatment for patients with pSPN, but the traditional radical resection of pancreatic malignant tumor is more traumatic, which is easy to cause postoperative pancreatic secretion insufficiency and high risk. At present, clinicians tend to take smaller surgical methods for patients with pSPN. Non-invasive pSPN is mainly enucleation of the tumor as a whole, and the prognosis is good, but the scope of resection of invasive pSPN is larger, and incomplete resection of the tumor may lead to recurrence and metastasis (7). Gao et al. (1). studies have shown that positive incisal margin increases the risk of postoperative pSPN recurrence, so accurate preoperative judgment of the invasiveness of pSPN is a key factor in making clinical operation plans. However, it is often difficult to obtain pathological results before operation. the pathological diagnosis of puncture biopsy is restricted by the quality and quantity of samples, which can’t accurately reflect the heterogeneity of tumor, and the operation of puncture biopsy may cause tumor cells to spread along the needle path. This makes it difficult and controversial for surgeons to choose the mode of operation. Because of its high popularization rate, convenience and few contraindications, CT has become the first choice for pancreatic diseases, the preoperative diagnosis and evaluation of invasiveness of pSPN depend to a large extent on the imaging features of tumors. Some relevant scholars have analyzed the relationship between imaging features and invasiveness of pSPN, but the imaging data of patients in multiple studies are not all analyzed by the same radiologist and concluded that there may be some subjective differences, and the results are not the same. It is controversial to predict the invasiveness of solid pseudopapillary tumor of the pancreas only from CT signs, so it is necessary to explore reliable features to evaluate tumor invasiveness before operation.

Radiomics technique using various automatically extracted data characterization algorithms converts images into a high dimensional mineable feature space (8–10). Numerous studies have applied the emerging radiomics technique to improve diagnostic, identification, prognostic, and predictive accuracy of cancer research (11–14). Some scholars also try to apply radiomics in pancreatic tumor studies, such as malignancy prediction (15), histopathologic characteristics discrimination (16), vascular invasion prediction (17), prognosis prediction (18), and radiogenomics for genetic status prediction (19). However, to the best of our knowledge, there is no literature that has determined whether a radiomics signature derived from CT images would enable superior prediction of invasive behavior in patients with pSPN.

Considering the radiomics feature could be extracted from the single cross section (two dimensional, 2D) or multi-slices (three dimensional, 3D) of the tumor in CT images, the reported radiomics-based pancreatic cancer studies have either applied 2D segmentation (20) or 3D whole-tumor segmentation (14, 21–25). However, whether to select 2D regions of interest (ROIs) or 3D ROIs still remains unclear for invasive behavior prediction in pSPN. In addition, the previous studies also have shown that there is controversy between 2D and 3D radiomics analysis in tumor diagnosis or prognosis (26–30).

In this work, we proposed a CT radiomics-based classification method by considering the performance of 3D or 2D segmentation and multiple CT imaging phases to discriminate invasiveness and non-invasiveness pSPN. The developed CT imaging signature might help treatment decision-making, especially the choice of operation.

## Materials and Methods

### Patient Selection

**Figure 1:**
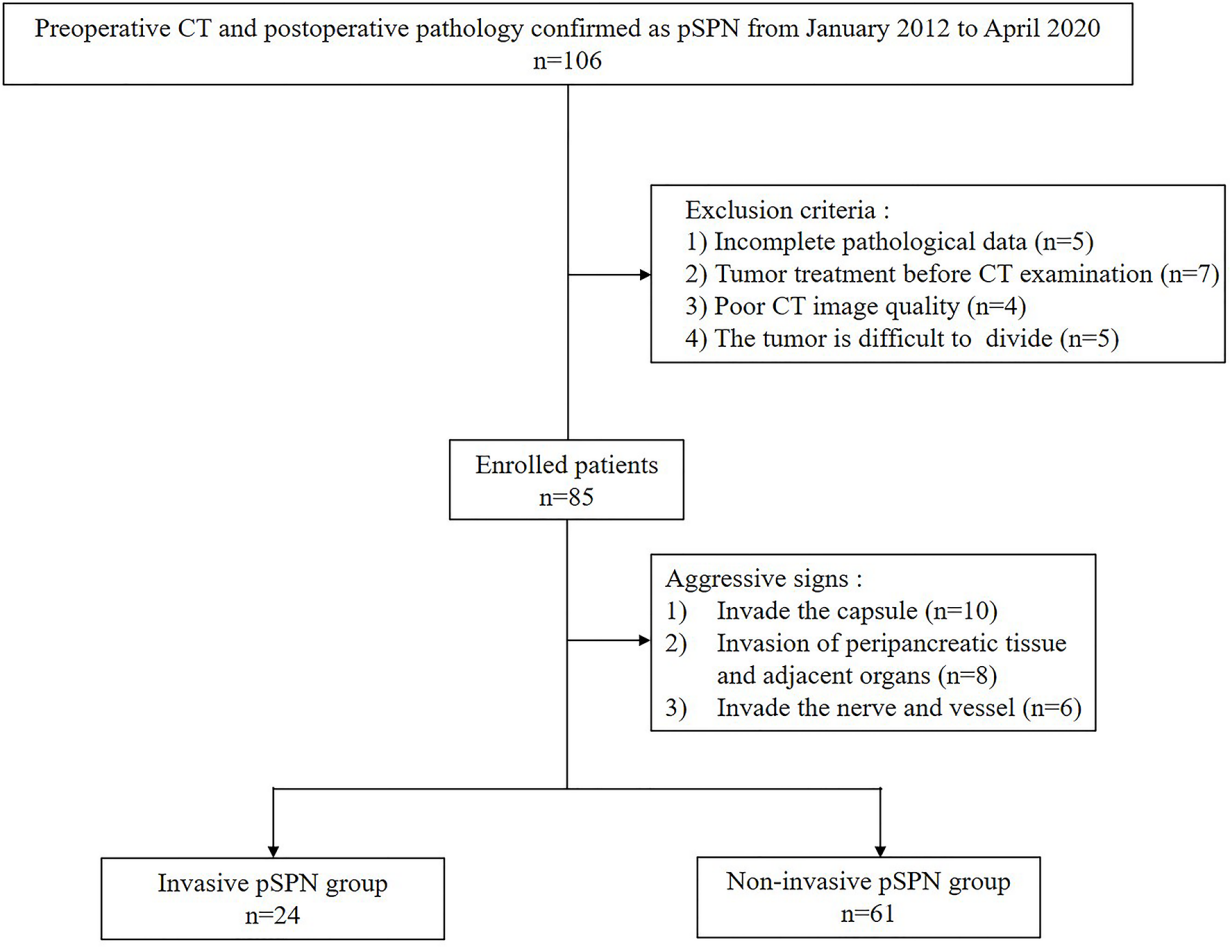
The patient enrollment workflow.

With institutional review board approval and waiver of the written informed consent, we retrospectively collected 85 patients with pSPN diagnosed by postoperative pathology from January 2012 to April 2020. The patient enrollment criteria included:1) the patient had no history of other malignant tumors before admission; 2) all patients with pSPN underwent surgery and the CT imaging data were complete; 3) abdominal CT plain scan and enhanced examination were performed within 30 days before operation; 4) the lesion covers at least 3 slices on CT cross section, and the maximum plane diameter is not less than 20mm. Exclusion criteria included:1) insufficient data of pathological diagnosis; 2) the patient had been punctured or treated with related tumor before CT examination; 3) poor CT image quality or lack of raw DICOM data; 4) there are a large number of ascites, pancreas or other lesions around the pancreas that cannot be divided. The flow chart of inclusion and exclusion of 85 patients is shown in **Figure 1**.

This cohort contained both screening and symptomatic cases (i.e., 48 and 37 cases respectively). There were 85 lumps on 85 cases, of which 24 tumor masses were invasive and 61 were non-invasive demonstrated by postoperative pathology. Patient characteristics in the invasive and non- invasive cohorts are given in **Table 1**.

**Table 1:** The clinical features of pancreatic solid pseudopapillary neoplasm.

Characteristics		Number of cases	Percentage
Age, mean ± SD (years)	32.12 ± 13.66		
Gender	Male	18	21.95%
	Female	64	78.05%
Clinical symptoms	Abdominal pain	43	52.43%
	Abdominal mass	4	4.88%
	Physical examination	35	42.68%

### CT Image Acquisition

All patients underwent contrast-enhanced CT scan and informed consent forms were signed before inspection. The CT scans were acquired with a 64-row CT scanner (Discovery CT 750 HD, GE Healthcare, Waukesha, WI, United States) or a dual source CT scanner (Somatom Definition Flash, S+iemens Healthineers, Forchheim, Germany). Conventional axial scanning was performed before and after an intravenous (i.v.) injection of nonionic iohexol (iopromide, 370 mg/mL, GE Medical Systems, 1.5 mL/kg and 3 mL/s) through a dual-head pump injector (Medrad, Warrendale, PA, United States). The scanning parameters were as follows: tube voltage, 120 kV; automatic mA technology is used for tube current; field of view (FOV), 500 mm; matrix, 512 × 512 mm; slice thickness, 0.625 mm to 5 mm; scan spacing, 0.625 mm to 5 mm. Finally, a 20-mL saline flush was performed at a rate of 3 mL/s. Using low-dose trigger technique, when the descending aorta reached 100 HU after injection of contrast medium, arterial phase images were collected 10 seconds later, and venous phase images were collected at intervals of 30 seconds.

### Image Process and Lesion ROI Segmentation

The CT images in arterial and venous phases were firstly resampled isotropically into 1 mm ×1 mm × 1 mm voxel size by using trilinear interpolation, to reduce the heterogeneity resulted from different scanner (24, 25). Then the CT images in respective phases were sequentially imported into A.K. software (Artificial Intelligence Kit, GE Healthcare, version 3.3.0) and the lesions were separately delineated in each imaging phase. 2D segmentation was realized by delineating around the tumor outline for the largest cross-sectional area in the CT axial plane. By conducting slice-by-slice delineation along with the tumor outer contour in the CT axial plane, 3D ROI was automatically merged. Each ROI was outlined by a radiologist (H.WP, 5 years of experience in abdominal imaging diagnosis) and supervised by a radiologist (L.P, 10 years of experience in abdominal imaging diagnosis). All the segmentations were finally accomplished with the consensus of these two radiologists. At the same time, thirty CT image sets were randomly and separately chosen from arterial and venous phase for assessing inter-observer repeatability of radiomics features. The ROIs were outlined by another radiologist (H.YJ, 5 years of experience in abdominal imaging diagnosis) and supervised by another radiologist (L.LM, 8 years of experience in abdominal imaging diagnosis). The features were then extracted and the features with intra-class correlation coefficient (ICC) greater than 0.75 were retained for further analysis, which meant a good feature reliability (31).

### Radiomics Feature Extraction

The radiomics features were automatically extracted by using Python package Pyradiomics (32). And before feature extraction, the CT values included in the ROI were discretized with binWidth = 25 HU (33). 1316 radiomics features were separately extracted from the delineated 2D or 3D ROIs in arterial and venous phases. There were 107 features extracted from the original images including: 32 first-order features (18 intensity statistical and 14 shape features). Among 75 textural features, there were 24 Gray Level Co‐occurrence Matrix (GLCM), 16 Gray Level Run Length Matrix (GLRLM), 16 Gray Level Size Zone Matrix (GLSZM), 14 Gray Level Dependence Matrix (GLDM) and 5 Neighboring Gray Tone Difference Matrix (NGTDM) features. By using transformed images, 1209 first-order and textural features were calculated, including 744 wavelet features based on level-1 wavelet decomposition images in three directions and 8 channels of LLL, HHH, LHL, LLH, HLL, HLH, HHL and LHH; 186 Laplacian of Gaussian (LoG) filtered features with sigma 2.0 mm and 3.0 mm and 279 features based on local binary pattern (LBP) filtered images including 2 sets of images based on 2-level spherical harmonics and 1 set of kurtosis image. For each transformed image, the same 6 kinds of features (93 features in total per image) were extracted including: 1) first-order features (18 intensity statistical features); 2) 24 Gray Level Co‐occurrence Matrix (GLCM) features; 3) 16 Gray Level Run Length Matrix (GLRLM) features; 4) 16 Gray Level Size Zone Matrix (GLSZM) features; 5) 14 Gray Level Dependence Matrix (GLDM) features; 6) 5 Neighboring Gray Tone Difference Matrix (NGTDM) features. Thirty CT image sets were randomly and separately chosen from arterial and venous phase for assessing inter-observer repeatability of radiomics features.

### Feature Selection and Model Construction

The radiomics features extracted from 2D and 3D ROIs in arterial and venous phases were firstly processed. The missing values were firstly replaced with median values. Then z-score normalization was used for standardization. As the class distribution of the original dataset was moderately imbalanced (invasive:24; non-invasive:61; ratio of 2.5), 200% data oversampling based on Synthetic Minority Oversampling Technique (SMOTE)was conducted to obtain equilibrium for class distribution (34, 35). The generated dataset (SMOTE dataset) involve 72 invasive and 96 noninvasive samples respectively (ratio of 1.3), which was used as training set for modeling.

The radiomics models were constructed separately based on 2D and 3D ROIs. While, the same feature selection and modeling methods were applied. The feature selection and final modeling procedure was performed in the SMOTE dataset as follows.

1. The features with agreement coefficient larger than 0.75 during the inter-observer consistency analysis were retained.
2. The features with relatively low variance less than 1.0 were excluded.
3. The features with less collinearity were retained by using correlation analysis at cut-value 0.7.
4. The features with significant difference (P<0.05) between invasive and noninvasive groups were selected by using univariate analysis (Mann-Whitney U test or t-test).
5. The least absolute shrinkage and selection operator (LASSO) logistic regression involving 10-fold cross validation was conducted to avoid overfitting (36). The maximum area under the curve (AUC) for model fitting among the 10-folds cross validation was applied to determine the lambda values, at which the remaining features with non-zero coefficients were retained.
6. The retained features after LASSO regression were finally involved into backward stepwise logistic regression with minimum AIC (Akaike Information Criterion) criteria to develop the regression radiomics model and radiomics-derived signature “Radscore” was derived by using the regression coefficients, which could be further transferred into probability by using sigmoid function P (Radscore) = 1/(1+exp(-Radscore)).

Hence, there were four basic radiomics models and corresponding Radscore derived, including the arterial phase model based on 2D ROIs (Radscore^AP_2D^), venous phase model based on 2D ROIs (Radscore^VP_2D^), arterial phase model based on 3D ROIs (Radscore^AP_3D^) and venous phase model based on 3D ROIs (Radscore^VP_3D^). Besides these four basic radiomics models, additional two combined models were also constructed, including the arterial-venous combined model based on 2D ROIs (Radscore^AP_VP_2D^) and arterial-venous combined model based on 3D ROIs (Radscore^AP_VP_3D^).

### Evaluation of Model Predictive Performance

The discrimination of the radiomics models were assessed by the receiver operating characteristic (ROC) curve analysis, and the area under the ROC curve (AUC), sensitivity, specificity and accuracy could be derived. In order to validate the constructed model based on the SMOTE dataset, the constructed single or combined model were applied in the original dataset. The regression coefficients and the model cut-off value (when Youden index reached the maximum) derived in the SMOTE dataset were applied in the original dataset. And the AUC, sensitivity, specificity and accuracy in the original dataset could be obtained to validate the model performance. Furthermore, the 1000-times bootstrap was used to assess the optimism and overall performance of radiomics models (37). To investigate the consistency of radiomics model for predicting invasiveness of pSPN in both SMOTE and original datasets, the calibration curves were plotted. Meanwhile, the decision curve analysis (DCA) was also used for assessment of the model clinical usefulness. In order to compare the ROC performance of each same kind of model between 2D and 3D ROIs, or to perform comparison between paired model from different phases, the Delong’s test was applied.

### Statistical Analysis

Statistical analysis was conducted by R software (version 3.5.3; http://www.r-project.org). Student’s t test or Mann-Whitney U test was used for continuous variables with normal or non-normal distribution (the Shapiro–Wilk test for assessing the normality of distribution) and the categorical variables were tested by Chi-square (or Fisher’s exact test). The Delong’s test was used for comparison of AUC between each paired model. The statistical significance levels were two-sided with P< 0.05. The following R packages were applied: “DMwR” for SMOTE oversampling; “findCorrelation” in “caret” package for correlation analysis; “glmnet” for logistic regression including LASSO regression algorithm; “pROC” for ROC analysis, and “rmda” for DCA analysis.

## Results

### Feature Selection and Radiomics Model Development

There were totally 6 radiomics models constructed based on 2D and 3D ROIs in arterial and venous imaging phase.

#### Arterial Phase Model Based on 3D ROI

By using inter-observer consistency analysis, 825 radiomics features with ICCs>0.75 were retained among 1316 features. After removing features with variance less than 1.0, 409 features were kept. Then, 25 features were retained after correlation analysis by using cut-value 0.7. Among 19 features selected by Mann-Whitney U test, 5 features were further removed by LASSO regression (**Figure S1**). Finally, 11 radiomics features were kept by backward stepwise logistic regression analysis (minimum AIC criteria), and the regression function deriving the Radscore^AP_3D^ was summarized in **Supplementary Equation (S1)**.

#### Venous Phase Model Based on 3D ROI

By using inter-observer consistency analysis, 810 radiomics features with ICCs>0.75 were retained among 1316 features. After removing features with variance less than 1.0, 385 features were kept. Then, 20 features were retained after correlation analysis by using cut-value 0.7. Among 17 features selected by Mann-Whitney U test, 13 features were retained after LASSO regression (**Figure S2**). Finally, 8 radiomics features were kept by backward stepwise logistic regression analysis (minimum AIC criteria), and the regression function deriving the Radscore^VP_3D^ was summarized in **Supplementary Equation (S2)**.

#### Arterial Phase Model Based on 2D ROI

By using inter-observer consistency analysis, 1059 radiomics features with ICCs>0.75 were retained among 1316 features. After removing features with variance less than 1.0, 475 features were kept. Then, 23 features were retained after correlation analysis by using cut-value 0.7. Among 12 features selected by Mann-Whitney U test, 8 features were retained after LASSO regression (**Figure S3**). Finally, 7 radiomics features were kept by minimum AIC criteria, and the regression function deriving the Radscore^AP_2D^ was summarized in **Supplementary Equation (S3)**.

#### Venous Phase Model Based on 2D ROI

By using inter-observer consistency analysis, 1126 radiomics features with ICCs>0.75 were retained among 1316 features. After removing features with variance less than 1.0, 517 features were kept. Then, 32 features were retained after Spearman correlation analysis by using cut-value 0.7. Among 12 features selected by Mann-Whitney U test, no feature was removed after LASSO regression (**Figure S4**). Finally, 7 radiomics features were kept by minimum AIC criteria, and the regression function deriving the Radscore^VP_2D^ was summarized in **Supplementary Equation (S4)**.

#### Arterial-Venous Combined Model Based on 3D ROI

The derived Radscore^AP_3D^ and Radscore^VP_3D^ were involved directly into multivariate logistic regression to construct the Arterial-Venous combined model based on 3D ROIs and the regression function deriving the Radscor^AP_VP_3D^ was summarized in **Supplementary Equation (S5)**.

#### Arterial-Venous Combined Model Based on 2D ROI

The derived Radscore^AP_2D^ and Radscore^VP_2D^ were involved directly into multivariate logistic regression to construct the Arterial-Venous combined model based on 2D ROIs and the regression function deriving the Radscor^AP_VP_2D^ was summarized in **Supplementary Equation (S6)**.

**Figure 2:**
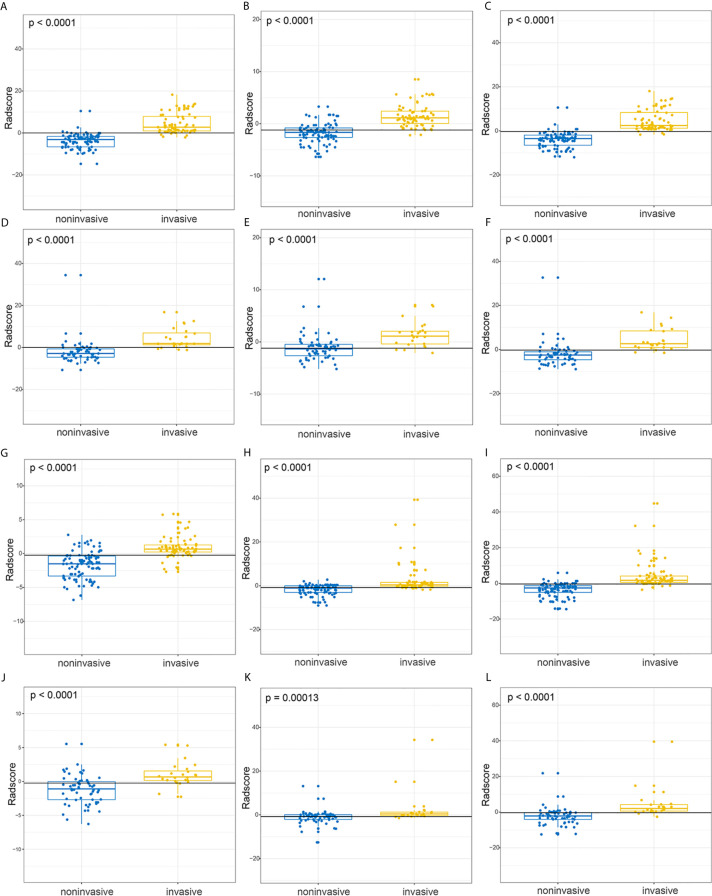
The boxplot for Radscores from 2D-domain and 3D-domain radiomics models and their statistical differences between invasive and noninvasive groups. **(A–C)** The respective distribution of Radscore^AP_3D^, Radscore^VP_3D^, Radscore^AP_VP_3D^ in the invasive (yellow) and noninvasive (blue) groups in the SMOTE dataset. **(D–F)** The respective distribution of Radscore^AP_3D^, Radscore^VP_3D^, Radscore^AP_VP_3D^ in the invasive (yellow) and noninvasive (blue) groups in the original dataset. **(G–I)** The respective distribution of Radscore^AP_2D^, Radscore^VP_2D^, Radscore^AP_VP_2D^ in the invasive (yellow) and noninvasive (blue) groups in the SMOTE dataset. **(J–L)** The respective distribution of Radscore^AP_2D^, Radscore^VP_2D^, Radscore^AP_VP_2D^ in the invasive (yellow) and noninvasive (blue) groups in the original dataset.

A statistically significant difference existed in Radscore^AP_3D^, Radscore^VP_3D^ were Radscor^AP_VP_3D^ between noninvasive and invasive in the original datasets with (-2.85(-4.66, -0.60) vs. 1.90(1.02, 7.25), p < 0.001), (-1.34(-2.65, -0.41) vs. 1.11(-0.44, 2.10), p < 0.001), (-2.51(-4.71, -1.02) vs. 2.63(0.92, 8.58), p < 0.001). Meanwhile, such significant difference also existed in Radscore^AP_3D^, Radscore^VP_3D^ were Radscor^AP_VP_3D^ between noninvasive and invasive in the original datasets with (-1.24 ± 2.12 vs. 1.01 ± 1.81, p < 0.001), (-0.79(-2.15, 0.09) vs. 0.38(-0.34, 1.25), p < 0.001), (-2.17(-4.51, -0.27) vs. 2.01(0.53, 4.24), p < 0.001). The distribution of each model’s Radscore in the invasive and noninvasive and the P values for the statistical difference analysis were also shown in **Figure 2** and their inset.

### Radiomics Model Performance

**Figure 3:**
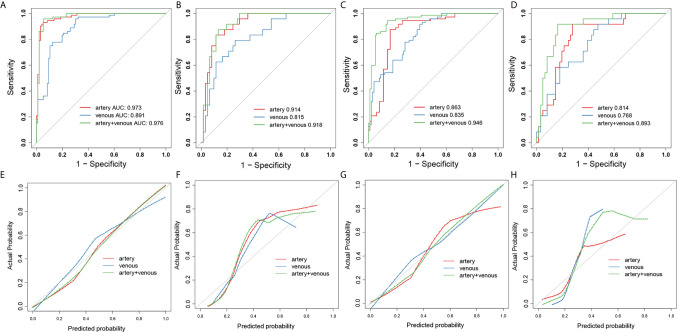
The ROC and calibration curves for 3D- and 2D- radiomics models. The ROC curves of artery-(red), venous- (blue) and combined artery-venous (green) radiomics models based on 3D ROIs in the SMOTE dataset **(A)** and original dataset **(B)**. The ROC curves of artery-(red), venous-(blue) and combined artery-venous (green) radiomics models based on 2D ROIs in the SMOTE dataset **(C)** and original dataset **(D)**. The calibration curves of artery-(red), venous-(blue) and combined artery-venous radiomics (green) models based on 3D ROIs in the SMOTE dataset **(E)** and original dataset **(F)**. The calibration curves of artery-(red), venous-(blue) and combined artery-venous (green) radiomics models based on 2D ROIs in the SMOTE dataset **(G)** and original dataset **(H)**.

The ROC analysis was used to evaluate the predictive performance of the constructed six models and the ROC curves for each model in SMOTE and original dataset were illustrated in **Figure 3**. The AUC, specificity, sensitivity, accuracy and the determined cut-value for each model performance in SMOTE dataset and the original dataset were summarized in **Tables 2** and **3**. Based on 3D ROIs, the arterial phase model has better performance than venous phase model and the arterial-venous combined model performed slightly better than the others. Based on 2D ROIs, the arterial phase model performed better than venous phase model, while the arterial-venous combined model performed better than any model constructed by independent imaging phase. In addition, the 3D arterial model performed better than the best arterial-venous combined 2D model. The Delong’s test result showed that the significant difference of model AUC existed in arterial models in original dataset (p = 0.019) while not in arterial-venous combined model (p=0.49) as comparing 2D and 3D segmentations (**Table S1**). In addition, the AUC of each selected 2D-based or 3D-based radiomics feature and their statistical differences between noninvasive and invasive groups were also summarized in the **Tables S2–S5**.

**Table 2:** Performance of 2D-domain radiomics model in predicting invasive behavior of pancreatic solid pseudopapillary neoplasm.

	SMOTE dataset			Original dataset		
	Artery	Venous	Artery +Venous	Artery	Venous	Artery +Venous
Threshold	-0.248	-0.819	-0.337	-0.248	-0.819	-0.337
AUC (95%CI)	0.863 (0.806-0.921)	0.835 (0.777-0.894)	0.946 (0.913-0.980)	0.814 (0.717-0.910)	0.768 (0.665-0.871)	0.893 (0.821-0.966)
Specificity	0.833	0.604	0.854	0.721	0.492	0.738
Sensitivity	0.875	0.917	0.944	0.875	0.917	0.917
Accuracy	0.851	0.738	0.893	0.765	0.612	0.788
NPV	0.899	0.906	0.953	0.936	0.938	0.957
PPV	0.797	0.635	0.829	0.553	0.415	0.579

**Table 3:** Performance of 3D-domain radiomics model in predicting invasive behavior of pancreatic solid pseudopapillary neoplasm.

	SMOTE dataset			Original dataset		
	Artery	Venous	Artery +Venous	Artery	Venous	Artery +Venous
Threshold	0.024	-1.195	-0.262	0.024	-1.195	-0.262
AUC (95%CI)	0.973 (0.950-0.996)	0.891 (0.843-0.939)	0.976 (0.953-0.998)	0.914 (0.854-0.974)	0.815 (0.718-0.912)	0.918 (0.860-0.976)
Specificity	0.948	0.698	0.948	0.820	0.525	0.803
Sensitivity	0.931	0.958	0.958	0.833	0.875	0.875
Accuracy	0.940	0.810	0.952	0.824	0.624	0.824
NPV	0.948	0.957	0.968	0.926	0.914	0.942
PPV	0.931	0.704	0.932	0.645	0.420	0.636

**Figure 4:**
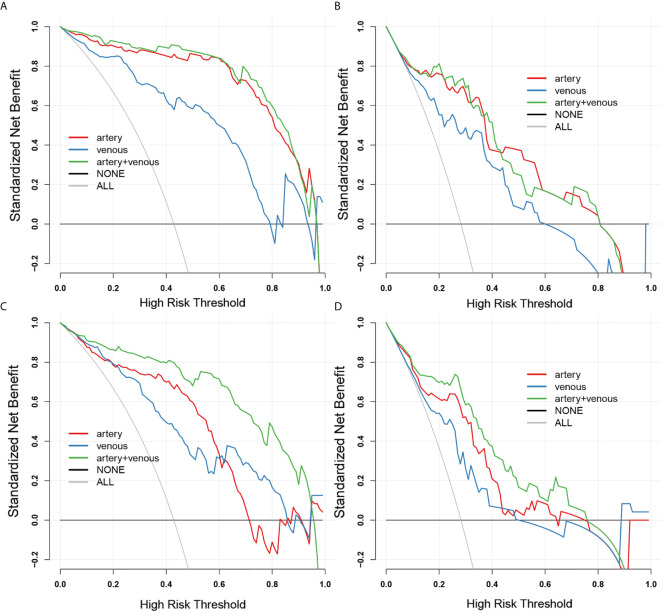
The decision curves for 2D-domain and 3D-domain radiomics models. **(A, B)** The decision curves for 3D-domain radiomics models in SMOTE dataset **(A)** and original dataset **(B)**. **(C, D)** The decision curves for 2D-domain radiomics models in SMOTE dataset **(C)** and original dataset **(D)**. The black horizontal line manifests no patients is invasive type (NONE) and the grey line manifests all patients are invasive type (ALL). The colored lines of each model respectively illustrate the net benefit brought to each patient based on artery-(red), venous-(blue) and combined artery-venous (green) radiomics models. The closer the decision curves to the black and gray curves, the lower the clinical decision net benefit of the model.

The arterial-venous combined models in both of 2D and 3D conditions showed a relatively good agreement between predicted and actual probability as shown by calibration curves in **Figure 4**. The overall performance of radiomics logistic regression model trained in the SMOTE dataset among 1000-times bootstrap were summarized in **Tables S6** and **S7**. The appearing frequency of each radiomics feature in the 3D and 2D logistic regression models during 1000-times bootstrap was respectively illustrated in **Figures S1** and **S2**. The respective optimism-corrected model’s AUC (3D arterial model: 0.928; 3D venous model: 0.832; 2D arterial model: 0.815; 2D venous model: 0.78) and their average optimism (3D arterial model: 0.045; 3D venous model: 0.059; 3D arterial model: 0.048; 3D venous model: 0.055) represents a relatively good reliability of the model established from the selected feature. In **Figure S2**, all of the features selected in the final 2D and 3D models appeared over 500 times during 1000-times bootstrap, which also reflected the reliability of the features. As shown by the DCA curves in **Figure 4**, in 3D models, the arterial model and arterial-venous combined model have wider range for risk threshold (0-0.9) than venous model to make model net benefit larger than 50%. And in 2D models, the arterial-venous combined model has wider range for risk threshold (0-0.6) than arterial or venous model alone to reach model net benefit exceed 50%.

## Discussion

In this retrospective study, we applied radiomics techniques to predict the invasiveness of pancreatic solid pseudopapillary neoplasm (pSPN), and established radiomics models to evaluate tumor invasiveness before operation based on CT images. The results showed that the 3D-domain radiomics features and 2D-domain features are potential predictors. Therefore, the classifier based on radiomics features could potentially provide a noninvasive and personalized management method for pSPN patients.

Previous studies mainly focused on the characteristic manifestations of CT and/or magnetic resonance imaging (38). However, it is difficult to identify whether pSPN is invasive before operation (39). Radiomic analysis has been proposed as a step towards realization of precision medicine by providing means to interrogate the spatial complexity of tumors *in vivo* (40). Therefore, we try to identify the invasiveness of pSPN by radiomics features and comprehensively consider the effectiveness of different contrast-enhanced phases and 2D or 3D segmentation.

The results showed that when using the single-phase-based radiomics features to predict the invasiveness of pSPN, the arterial phase features were more effective than the venous phase features and there was no statistically significant model’s AUC improvement in the original dataset when combining arterial and venous phase compared with sole arterial-phase model. The arterial phase enhancement characteristics of the tumor reflected the characteristics of tumor blood supply and functional capillaries, and the invasive tumor had more obvious blood supply. On the one hand, angiogenesis is closely related to the occurrence, development and prognosis of the tumor, but there are complex substances such as collagen and hyaluronic acid in the cell stroma. During the venous phase, the interstitial components of the tumor will also be enhanced because of the inflow of the contrast medium. As a result, the vascular enhancement of the tumor in the venous phase is not obvious. The arterial phase images can only enhance the blood vessels of the tumor, because the interstitial components will not be enhanced. Thus the arterial phase images can better reflect the characteristics of blood supply in the tumor and reflect the characteristics of the tumor more accurately. In some related studies of pancreatic tumors, Kwon et al. (41) especially emphasized the role of enhanced MRI in arterial phase in the study of the differentiating focal autoimmune pancreatitis and pancreatic ductal adenocarcinoma. Corwin et al. (42). reported that the greater attenuation differential between lesions and normal pancreas during the arterial phase compared to venous. Bian et al. (23). also reported a significant positive association between the arterial-phase-based radiomics Radscore and the risk of LN metastasis in pancreatic ductal adenocarcinoma. In addition, it was also the arterial-phase model could have higher multivariable AUC in predicting malignancy and invasive pathological status of pancreatic intraductal papillary mucinous neoplasms compared with that of venous-phase model (14). On the other hand, the invasive biological behavior of invasive pSPN is more prone to vascular invasion, and peripheral blood vessels are easily involved, especially the venous wall is thin and the pressure is low, and the tumor is easy to cause stenosis of venous branches. If the formation of microtumor thrombus can lead to obstruction of venous branches in microcirculation and obstruction of blood flow, resulting in an increase of compensatory blood supply of arteries. These results might reflect the predictive capability of the arterial-phase-based radiomics features in predicting tumor invasiveness.

Most prior studies have employed either a single slice or whole tumor to extract features for radiomics analysis. However, the actual effect of using features extracted from 2D slices or 3D volumes varies. In a colorectal cancer prognosis study, it has shown that the CT feature extracted from 3D segmentation was superior to the largest cross-sectional segmentation to predict the survival rate (16). Some scholar reports also showed that the 2D and 3D segmentation possessed similar capability in clinical outcome prediction, such as the determination of pathological feature or prognosis in hepatic metastatic colorectal cancer (CRC) (28) and prediction of axillary lymph node metastasis in breast cancer (29). However, it has also been reported that 2D CT radiomics features performed better in prognosis prediction in lung cancer (30). These studies show that the results of 2D or 3D radiological analysis need to be further studied by different patient cohorts and tasks. Our study indicated that the AUC value of 3D-based model is greater than that of 2D-based model in both SMOTE dataset and original dataset. Except for that the AUC value of 2D venous model is 0.768, the AUC values of all models are all greater than 0.80. In the original dataset, the ROC comparison of 2D-based and 3D-based arterial-phase model was statistically significant. Multi-slices (3D) analysis covers the entire tumor volume and can better depict spatial heterogeneity than using a single tumor slice (2D) (27).

In terms of feature contribution to each 2D or 3D model in the current study, most of the selected features were filtered or transformed first-order or texture features. It might indicate that the distinguishment between the noninvasive and invasive pSPN might need the emphasized features in the spatial or frequency domains. It could be found that there existed some overlaps for selected 2D and 3D feature types in the artery-phase models, including the RunVariance (GLRLM), Median (First order), Kurtosis (First order) and DependenceVariance (GLDM), which represent similar tendency between invasive and noninvasive groups. However, the 3D features’ AUC located in the range of 0.58-0.72, which were slightly higher than that of 2D features (AUC from 0.58 to 0.66). And the features that not involved in the 2D model, such as wavelet.LLL_firstorder_InterquartileRange, original_firstorder_Skewness, and wavelet.HHL_glrlm_LongRunLowGrayLevelEmphasis could even have acceptable AUC values larger than 0.65. First-order statistics features describe the distribution of voxel intensity in the ROI region. GLDM texture features mainly describe the gray level dependence between voxels. GLRLM quantifies the length of consecutive pixels that have the same grey level. Three-dimensional ROI includes the whole lesion and does not avoid cystic necrosis and calcification in the tumor. It could have more chances compared with 2D cross-section to extract intensity or texture details distributed in 3D space or multiple 3D directions and select the representative features describing the internal structure or pathological heterogeneity of tumor which are closely related to invasiveness. This might indicate that intensity or texture details distributed in 3D space or multiple 3D directions could be extracted from the entire volume rather than those from the single slice. In the current study, 3D features can better predict invasiveness from a global point of view.

Thus, the noninvasive radiomics signature could serve as a more convenient biomarker for the prediction of invasive behavior in pSPN. To justify the clinical usefulness, decision curve analysis was applied in this study to confirm the predictive value of the imaging group model. This novel method offers insight into clinical consequences on the basis of threshold probability, from which the net benefit could be derived.

There are still some limitations in this study. Firstly, the disease is rare, all available data have been collected, but the sample size in this study was small and had some class imbalance with noninvasive-to-invasive sample ratio of 2.5. The data were augmented and balanced by SMOTE method into noninvasive-to-invasive sample ratio of 1.3 and used as the training set of the model, which was further internally validated in the original dataset to test the model performance as much as possible. Secondly, it was not combined with clinical data and pathological immunohistochemical results. A study of radiomics and clinical data is needed in the future to further reveal the biological or clinical meanings or association for the radiomics features, which still could not be well explained in the current study. In addition, this is a single-center study, and we are working to further evaluate our model in a bigger dataset that may come from multiple centers and multiple imaging schemes.

## Conclusion

In conclusion, a radiomics method based on CT imaging data was developed and validated as a potential method for predicting invasiveness of pSPN before the operation in our study. Radiomics model showed encouraging performance and is expected to provide an intelligent, non-invasive diagnostic tool for predicting the invasiveness of pSPN. Further research is needed to explore the relationship between radiomics features and clinicopathological index and establish more generalized prediction models.

## Data Availability Statement

The original contributions presented in the study are included in the article/**Supplementary Material**. Further inquiries can be directed to the corresponding author.

## Author Contributions

W-pH and PL: designed the research. L-mL and W-pH: performed the research. Y-jH: collected the data. S-yL and W-pH: analyzed the data and wrote the paper. J-bG: reviewed the paper. All authors contributed to the article and approved the submitted version.

## Funding

This work was supported by the National Natural and Science Fund of China (No. 81971615).

## Conflict of Interest

Author S-yL was employed by General Electric Company (GE) Healthcare.

The remaining authors declare that the research was conducted in the absence of any commercial or financial relationships that could be construed as a potential conflict of interest.
