# Supplementary material for: Development of CT-Based Imaging Signature for Preoperative Prediction of Invasive Behavior in Pancreatic Solid Pseudopapillary Neoplasm
Source: Front Oncol. 2021 May 17;11:677814. doi: 10.3389/fonc.2021.677814 (PMC8166224; doi:10.3389/fonc.2021.677814)
Supplement: Supplementary file 1 [file DataSheet_1.docx]

***Supplementary Material***

**Supplementary Method**

**The SMOTE method and the model performance based on different SMOTE parameters**

The SMOTE algorithm was used to synthesize more balanced data in the current study and the SMOTE process was realized by using R function of “SMOTE” in “DMwR” package (R3.5.3 version).

The detail about the SMOTE algorithm was described as follows:

Supposing that there was N1 samples (minority) while N2 samples (majority) among the original dataset, the SMOTE dataset could be synthesized at the given oversampling ratio a(%) and undersampling ratio b (%) based on K-nearest algorithm at a given K value. In the current study, the parameters were:N1 = 24, N2 = 61, a = b = 200, K = 5.

Firstly, the minority samples (number: N1) were augmented based on K-nearest algorithm (K = 5) to obtain an oversampled minority samples with number of N1^minority^ = N1+a×N1/100. In the current study, the N1^minority^= 24+200×24/100=72.

Secondly, the majority samples (number: N2) could be undersampled at ratio of b (%) to obtain a final sample size: N2^major^= (b/100)×(a×N1/100). In the current study, the N2^major^= (200/100)×(200×24/100)=96.

The final SMOTE dataset included N^total^ = (N1^minority^ + N2^major^) samples. In the current study, the N^total^ = (72 + 96) = 168.

The parameters a and b in the SMOTE algorithm could change the noninvasiveness-to-invasiveness ratio in the dataset, which is important for modeling. Therefore, following the reviewer’s valuable suggestion, we tried to set different a and b values resulting in different noninvasiveness-to-invasiveness ratio and assessed the model performance including Model^AP_3D^, Model^VP_3D^, Model^AP_2D^ and Model^VP_2D^. Here, we explored a ratio of 2 (a = 200, b = 300) and ratio of 1.33 (a = b = 200, which was used in the current study). In addition, the corresponding models constructed based on original dataset (non-SMOTE) were also evaluated. The models’ apparent AUC and their performance in the test dataset and overoptimism during 1000-times bootstrapping were summarized in Table S6 and Table S7.

It could be found that different noninvasiveness-to-invasiveness ratio could influence the retained feature number and model performance. Firstly, the model performance could be enhanced by using balanced data which was synthesized from SMOTE algorithm. Secondly, when applying the SMOTE parameters which result in the noninvasiveness-to-invasiveness ratio much closer to 1:1 (e.g. the ratio of 1.33 used in the current study), the model overoptimism could be decreased compared with the higher ratio. In addition, the training dataset with more balanced ratio could have fewer retained features which further help avoid model overfitting.

**Supplementary Equation**

**S1**＝－2.21＋2.65×wavelet. LLL_firstorder_InterquartileRange＋3.29×wavelet. HLL_gldm_Dependencevariance＋1.44×original_firstorder_Skewness－1.82×log.sigma.2.0.mm.3D_glrlm_RunVariance－1.32×log.sigma.2.0.mm.3D_firstorder_Median＋1.01×wavelet. LHL_firstorder_Kurtosis－1.48×wavelet. HHL_glrlm_LongRunLowGrayLevelEmphasis＋2.14×wavelet. HLL_ngtdm_Busyness－2.51×wavelet. LHL_gldm_Dependencevariance－1.32×log.sigma.2.0.mm.3D_firstorder_Skewness＋2.62×original_gldm_Dependencevariance

**S2**＝－0.42－2.35×wavelet. LLH_firstorder_Skewness＋0.63×wavelet. HLL_gldm_Dependencevariance＋0.85×wavelet. LLL_firstorder_InterquartileRange＋1.48×wavelet. LLL_firstorder_Skewness－0.66×log.sigma.2.0.mm.3D_firstorder_Median－0.83×log.sigma.2.0.mm.3D_firstorder_Kurtosis－0.69×original_glrlm_RunVariance－1.41×wavelet. LLH_gldm_Dependencevariance

**S3**＝－0.61－1.35×log.sigma.2.0.mm.3D_glrlm_RunVariance＋0.42×wavelet. HLH_gldm_LargeDependenceEmphasis＋0.69×wavelet. LLH_firstorder_Median＋0.85×log.sigma.3.0.mm.3D_firstorder_90Percentile－0.55×original_glrlm_RunVariance＋0.74×lbp.3D.k_firstorder_Kurtosis＋1.55×lbp.3D.m2_gldm_LargeDependenceLowGrayLevelEmphasis

**S4**＝－0.05＋0.37×wavelet. LLL_firstorder_InterquartileRange＋5.81×wavelet. LHL_glszm-LargeAreaLowGrayLevelEmphasis＋1.01×wavelet. LLL_gldm_LargeDependenceLowGrayLevelEmphasis－1.38×wavelet. LHL_gldm_LargeDependenceLowGrayLevelEmphasis－0.51×log.sigma.3.0.mm.3D_glrlm_LongRunLowGrayLevelEmphasis－3.18×original_glrlm_RunVariance＋1.13×wavelet. HLL_gldm_LargeDependenceLowGrayLevelEmphasis

**S5**＝0.049+ 0.841×S1+ 0.535×S2

**S6**＝0.355+ 1.252×artery+1.109×venous

**Table S1** ROC comparison in predicting invasive behavior of pancreatic solid pseudopapillary neoplasm

|  | SMOTE dataset | | original dataset | |
| --- | --- | --- | --- | --- |
| 2D | Statistics | *P* value | Statistics | *P* value |
| Artery vs Venous | 0.672 | 0.501 | 0.633 | 0.527 |
| Artery vs Artery +Venous | -3.289 | 0.001 | -1.930 | 0.054 |
| Venous vs Artery +Venous | -4.442 | ＜0.001 | -2.907 | 0.004 |
| 3D | Statistics | *P* value | Statistics | *P* value |
| Artery vs Venous | 3.541 | ＜0.001 | 2.171 | 0.030 |
| Artery vs Artery +Venous | -0.673 | 0.501 | -0.262 | 0.793 |
| Venous vs Artery +Venous | -4.010 | ＜0.001 | -2.773 | 0.006 |
| 2D vs 3D | Statistics | *P* value | Statistics | *P* value |
| Artery | 4.027 | ＜0.001 | 2.349 | 0.019 |
| Venous | 1.721 | 0.085 | 0.682 | 0.495 |
| Artery +Venous | 1.889 | 0.059 | 0.689 | 0.491 |

**Table** **S2** The statistical difference of each selected radiomics feature between invasive and noninvasive groups and their AUC for invasive prediction in arterial phase model based on 3D ROI of the original dataset

| Variable | Sample | AUC | Noninvasive | Invasive | Statistics | *P* value^c^ |
| --- | --- | --- | --- | --- | --- | --- |
| wavelet.LLL_firstorder_InterquartileRange | 85 | 0.724 | -0.28(-0.45, -0.10)^a^ | 0.16(-0.23, 0.66) | -3.202 | 0.001 |
| wavelet.HLL_gldm_DependenceVariance | 85 | 0.688 | -0.18(-0.73, 0.15) | 0.19(-0.31, 1.24) | -2.680 | 0.007 |
| original_firstorder_Skewness | 85 | 0.658 | -0.18(-0.31, -0.01) | -0.02(-0.23, 1.49) | -2.255 | 0.024 |
| log.sigma.2.0.mm.3D_glrlm-RunVariance | 85 | 0.636 | 0.10(-0.40, 0.47) | -0.28(-1.00, 0.15) | 1.943 | 0.052 |
| log.sigma.2.0.mm.3D_firstorder_Median | 85 | 0.571 | -0.11(-0.33, 0.11) | -0.20(-0.38, 0.12) | 1.02 | 0.308 |
| wavelet.LHL_firstorder_Kurtosis | 85 | 0.653 | -0.44(-0.47, -0.21) | -0.26(-0.46, 1.08) | -2.192 | 0.028 |
| wavelet.HHL_glrlm_LongRunLowGrayLevelEmphasis | 85 | 0.678 | -0.43(-0.64, 1.05) | -0.60(-0.74, -0.36) | 2.538 | 0.011 |
| wavelet.HLL_ngtdm_Busyness | 85 | 0.615 | -0.39(-0.52, 0.37) | -0.61(-0.70, -0.06) | 1.64 | 0.101 |
| wavelet.LHL_gldm_DependenceVariance | 85 | 0.577 | -0.10(-0.48, 0.26) | 0.13(-0.45, 0.72) | -1.093 | 0.274 |
| log.sigma.2.0.mm.3D_firstorder_Skewness | 85 | 0.576 | 0.50(-0.47, 0.77) | -0.17(-0.87, 0.65) | 1.084 | 0.279 |
| original_gldm_DependenceVariance | 85 | 0.587 | 0.07±0.93^b^ | -0.17±1.16 | 0.976 | 0.332 |

a. The variables without normal distribution were depicted by median (interquartile range, IQR)

b. The variables with normal distribution were depicted by mean ± SD.

c. Statistically significant level: P < 0.05

**Table** **S3** The statistical difference of each selected radiomics feature between invasive and noninvasive groups and their AUC for invasive prediction in venous phase model based on 3D ROI of the original dataset

| Variable | Sample | AUC | Noninvasive | Invasive | Statistics | *P* value^b^ |
| --- | --- | --- | --- | --- | --- | --- |
| wavelet.LLH_firstorder_Skewness | 85 | 0.726 | 0.40(0.21, 0.42)^a^ | 0.19(-0.62, 0.38) | 3.236 | 0.001 |
| wavelet.HLL_gldm_DependenceVariance | 85 | 0.678 | -0.12(-0.81, 0.28) | 0.28(-0.32, 0.84) | -2.538 | 0.011 |
| wavelet.LLL_firstorder_InterquartileRange | 85 | 0.618 | -0.23(-0.70, 0.20) | 0.08(-0.41, 0.80) | -1.684 | 0.092 |
| wavelet.LLL_firstorder_Skewness | 85 | 0.609 | -0.31(-0.50, 0.04) | -0.13(-0.42, 1.44) | -1.552 | 0.121 |
| log.sigma.2.0.mm.3D_firstorder_Median | 85 | 0.558 | -0.08(-0.35, 0.14) | -0.15(-0.40, 0.02) | 0.825 | 0.409 |
| log.sigma.2.0.mm.3D_firstorder_Kurtosis | 85 | 0.576 | -0.59(-0.67, 0.20) | -0.20(-0.63, 0.27) | -1.084 | 0.279 |
| original_glrlm_RunVariance | 85 | 0.593 | -0.31(-0.49, 0.20) | -0.42(-0.54, -0.22) | 1.328 | 0.184 |
| wavelet.LLH_gldm_DependenceVariance | 85 | 0.605 | -0.35(-0.75, 0.43) | -0.23(-0.56, 1.49) | -1.494 | 0.135 |

a. The variables without normal distribution were depicted by median (interquartile range, IQR)

b. Statistically significant level: P < 0.05

**Table** **S4** The statistical difference of each selected radiomics feature between invasive and noninvasive groups and their AUC for invasive prediction in arterial phase model based on 2D ROI of the original dataset

| Variable | Sample | AUC | Noninvasive | Invasive | Statistics | *P* value^c^ |
| --- | --- | --- | --- | --- | --- | --- |
| log.sigma.2.0.mm.3D_glrlm_RunVariance | 85 | 0.661 | 0.14±1.03^a^ | -0.35±0.84 | 2.078 | 0.041 |
| wavelet.HLH_gldm_LargeDependenceEmphasis | 85 | 0.586 | -0.11(-0.85, 0.65)^b^ | 0.52(-0.34, 0.74) | -1.230 | 0.219 |
| wavelet.LLH_firstorder_Median | 85 | 0.596 | -0.04(-0.34, 0.07) | -0.02(-0.13, 0.38) | -1.367 | 0.172 |
| log.sigma.3.0.mm.3D_firstorder_90Percentile | 85 | 0.619 | -0.26(-0.70, 0.18) | 0.11(-0.46, 0.77) | -1.699 | 0.089 |
| original_glrlm_RunVariance | 85 | 0.611 | -0.26(-0.41, 0.02) | -0.34(-0.48, -0.15) | 1.591 | 0.112 |
| lbp.3D.k_firstorder_Kurtosis | 85 | 0.643 | -0.28(-0.80, 0.19) | 0.04(-0.42, 1.07) | -2.040 | 0.041 |
| lbp.3D.m2_gldm_LargeDependenceLowGrayLevelEmphasis | 85 | 0.617 | 0.16(-0.63, 0.69) | 0.53(-0.24, 0.93) | -1.679 | 0.093 |

a. The variables with normal distribution were depicted by mean ± SD.

b. The variables without normal distribution were depicted by median (interquartile range, IQR)

c. Statistically significant level: P < 0.05

**Table** **S5** The statistical difference of each selected radiomics feature between invasive and noninvasive groups and their AUC for invasive prediction in venous phase model based on 2D ROI of the original dataset

| Variable | Sample | AUC | Noninvasive | Invasive | Statistics | *P* value^b^ |
| --- | --- | --- | --- | --- | --- | --- |
| wavelet.LLL_firstorder_InterquartileRange | 85 | 0.595 | -0.14(-0.33, 0.02)^a^ | -0.10(-0.24, 0.11) | -1.357 | 0.175 |
| wavelet.LHL_glszm_LargeAreaLowGrayLevelEmphasis | 85 | 0.670 | -0.29(-0.31, -0.10) | -0.32(-0.32, -0.23) | 2.431 | 0.015 |
| wavelet.LLL_gldm_SmallDependenceHighGrayLevelEmphasis | 85 | 0.629 | -0.21(-0.23, -0.14) | -0.19(-0.22, 0.05) | -1.845 | 0.065 |
| wavelet.LHL_gldm_LargeDependenceLowGrayLevelEmphasis | 85 | 0.656 | -0.24(-0.56, 0.56) | -0.53(-0.94, 0.20) | 2.236 | 0.025 |
| log.sigma.3.0.mm.3D_glrlm_LongRunLowGrayLevelEmphasis | 85 | 0.595 | -0.06(-0.83, 0.80) | -0.65(-0.86, 0.20) | 1.357 | 0.175 |
| original_glrlm_RunVariance | 85 | 0.620 | -0.35(-0.58, 0.28) | -0.48(-0.66, -0.12) | 1.708 | 0.088 |
| wavelet.HLL_gldm_LargeDependenceLowGrayLevelEmphasis | 85 | 0.613 | -0.02(-0.75, 0.38) | -0.29(-1.08, 0.08) | 1.621 | 0.105 |

a. The variables without normal distribution were depicted by median (interquartile range, IQR)

b. Statistically significant level: P < 0.05

**Table S6** 1000-times Bootstrap estimate of the area under the ROC curve and the model optimism estimation for the radiomics models constructed based on 200% SMOTE algorithm.

| **Index** | **Model** | **Apparent AUC^a^** | **AUC Bootstrap-Train^b^**  **(mean, 95%CI)** | **AUC Bootstrap-Test^c^**  **(mean, 95%CI)** | **Average optimism^d^** | **Optimism-corrected AUC^e^** |
| --- | --- | --- | --- | --- | --- | --- |
| **1** | **Model^AP_3D^** | 0.973 | 0.976(0.975-0.977) | 0.931(0.929-0.933) | 0.045 | 0.928 |
| **2** | **Model^VP_3D^** | 0.891 | 0.905(0.903-0.906) | 0.845(0.843-0.848) | 0.059 | 0.832 |
| **3** | **Model^AP_2D^** | 0.863 | 0.870(0.868-0.872) | 0.822(0.819-0.825) | 0.048 | 0.815 |
| **4** | **Model^VP_2D^** | 0.835 | 0.848(0.846-0.850) | 0.793(0.790-0.796) | 0.055 | 0.78 |

Note:

1. The AUC of predicting model developed in SMOTE dataset
2. The averaged model performance in the resampled training set after 1000-times bootstrap.
3. The averaged model performance in the “out-of-bag” test set after 1000-times bootstrap.
4. The model’s averaged optimism as the difference between the bootstrap training set AUC and the test AUC
5. The corrected AUC by subtracting the average optimism from the apparent AUC.

**Table S7** The model performance based on different noninvasiveness-to-invasiveness ratio and sample size of dataset.

| **Model** | **Ratio = 2.54 (original)**  **sample size = 85** | | | | **Ratio = 2 (SMOTE a=200, b=300)**  **Sample size = 216** | | | | **Ratio = 1.33 (SMOTE a=200, b=200)**  **Sample size = 168** | | | |
| --- | --- | --- | --- | --- | --- | --- | --- | --- | --- | --- | --- | --- |
|  | **AUC_train^a^** | **AUC_ori^b^** | **AUC_test^c^** | **Overopt^d^** | **AUC_train^a^** | **AUC_ori^b^** | **AUC_test^c^** | **Overopt^d^** | **AUC_train^a^** | **AUC_ori^b^** | **AUC_test^c^** | **Overopt^d^** |
| **Model^AP_3D^** | 0.76(0.642-0.877) (2 features) | - | 0.699(0.692-0.705) | 0.057 | 0.939(0.907-0.971) (12 features) | 0.922(0.861-0.983) | 0.897(0.895-0.899) | 0.055 | 0.973(0.950-0.995) (11 features) | 0.914(0.854-0.974) | 0.931(0.929-0.933) | 0.045 |
| **Model^VP_3D^** | 0.695(0.574-0.817) (2 features) | - | 0.625(0.619-0.631) | 0.056 | 0.892(0.847-0.936) (12 features) | 0.844(0.749-0.939) | 0.834(0.831-0.839) | 0.071 | 0.891(0.843-0.939) (8 features) | 0.815(0.718-0.912) | 0.845(0.843-0.848) | 0.059 |
| **Model^AP_2D^** | 0.680(0.552-0.808) (2 features) | - | 0.613(0.608-0.619) | 0.061 | 0.869(0.822-0.916) (11 features) | 0.833(0.748-0.917) | 0.813(0.811-0.816) | 0.076 | 0.863(0.806-0.921) (7 features) | 0.813(0.717-0.91) | 0.822(0.819-0.825) | 0.048 |
| **Model^VP_2D^** | 0.805(0.697-0.912) (4 features) | - | 0.711(0.705-0.717) | 0.106 | 0.869(0.821-0.917) (8 features) | 0.825(0.731-0.920) | 0.822(0.819-0.824) | 0.058 | 0.835(0.777-0.894) (7 features) | 0.768(0.665-0.871) | 0.793(0.790-0.796) | 0.055 |

1. AUC_train: the apparent AUC from the training set, which is the original dataset for non-SMOTE condition and the SMOTE-augmented dataset for SMOTE condition.
2. AUC_ori: the model’s AUC performance validated in the original dataset. The model is constructed based on the SMOTE-augmented dataset.
3. AUC_test: the model’s AUC performance validated in the out-of-bag test dataset during 1000-times bootstrapping. The model is constructed based on the SMOTE-augmented dataset.
4. Overopt: the model’s averaged optimism as the difference between the bootstrap training set AUC and the test AUC during 1000-times bootstrapping.


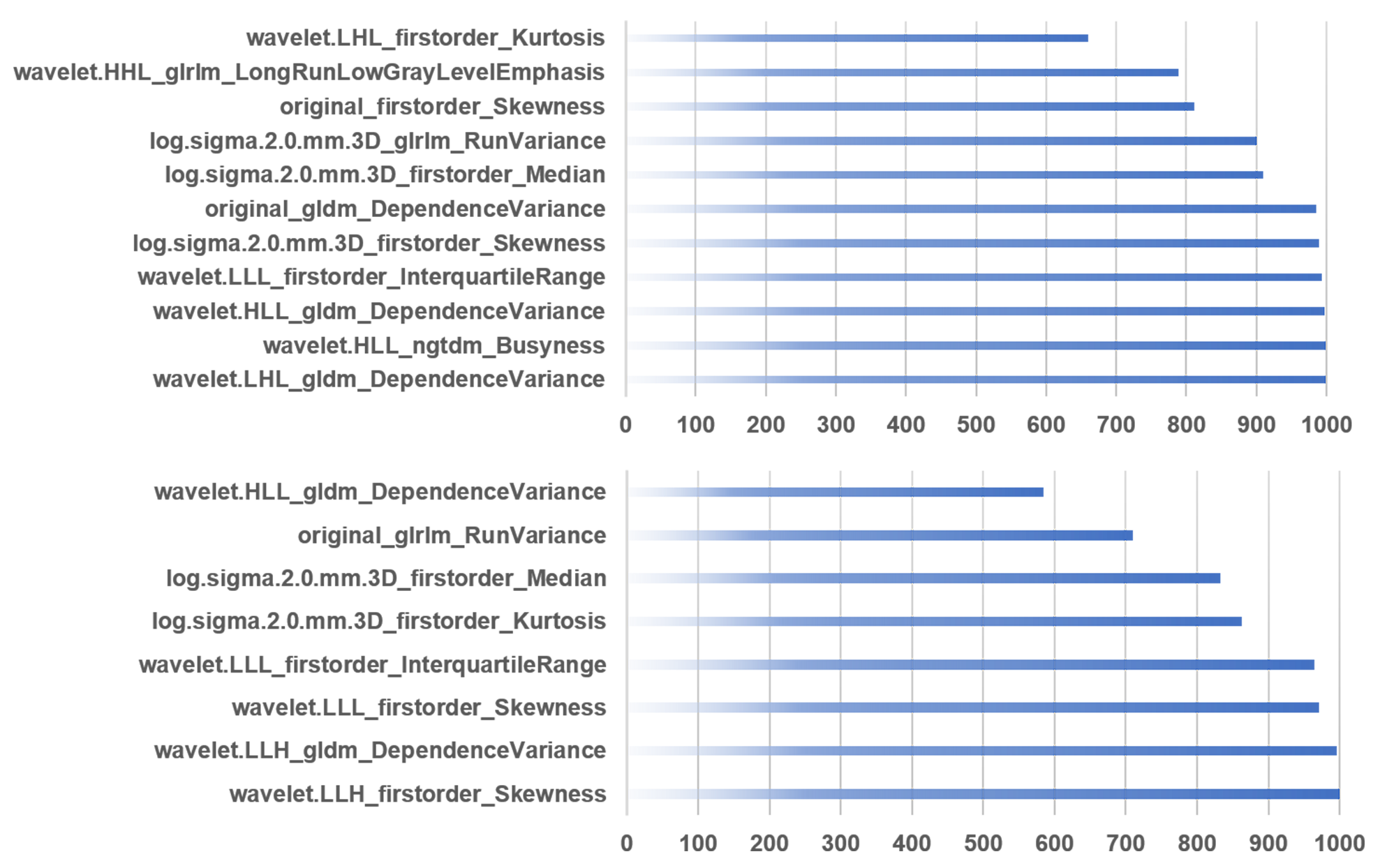


**Figure S1** The appearing frequency of radiomics features among 1000-times bootstrap in 3D artery model (top) and 3D venous model (bottom).


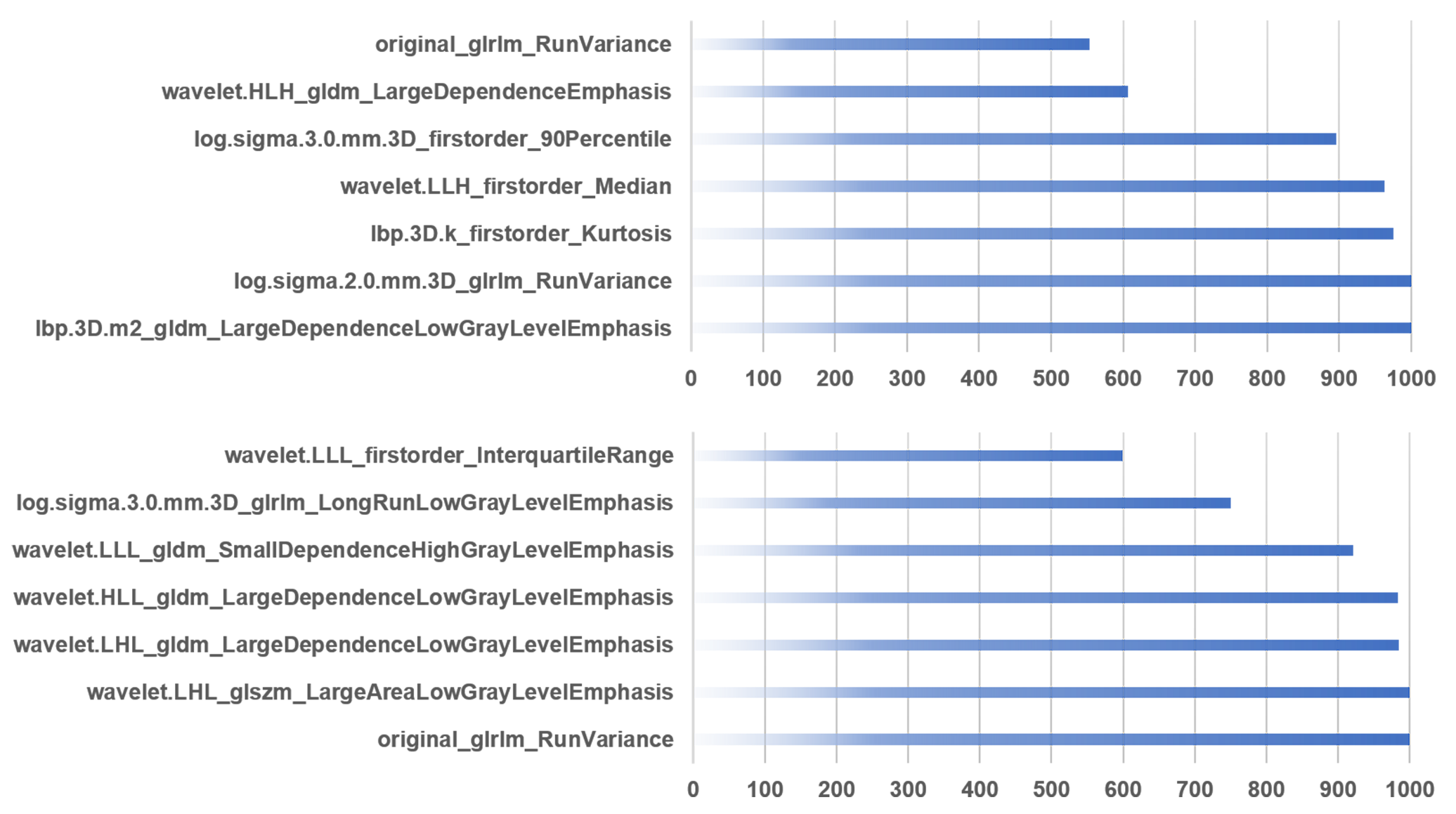


**Figure S2** The appearing frequency of radiomics features among 1000-times bootstrap in 2D artery model (top) and 2D venous model (bottom).
